# Alcohol intoxication and its influence on road traffic accidents: a hospital based study from Pondicherry, India

**DOI:** 10.1038/s41598-026-43509-5

**Published:** 2026-04-08

**Authors:** Arun Prakash K.S., Mohan S.

**Affiliations:** Present Address: Department of Forensic Medicine and Toxicology, SRM Medical college Hospital and Research Centre, Kattankulathur Campus, Chengalpattu, Kattankulathur, 603203 Tamil Nadu India

**Keywords:** Diseases, Health care, Health occupations, Medical research, Risk factors

## Abstract

**Background:** Driving under the influence of alcohol has caused a substantial number of traffic accidents. Alcohol consumption is a well-known contributor to road traffic injuries worldwide. Pondicherry is a city with a high and rising rate of road traffic-related injuries. It has generally been regarded as a region with high alcohol consumption.

**Aims: ** To examine the relationship between alcohol consumption and road traffic accidents.

**Methods:** This was a cross-sectional hospital-based study. From May 25, 2018, to August 22, 2018, treated in emergency rooms were analysed. A survey with a semi structured format was administered. During the study period, 329 patients involved in motor vehicle accidents were interviewed. The data were collected in ODK, exported to a spreadsheet in Microsoft Excel, and analysed via SPSS version 16. Various study variables are represented in the results as percentages. The chi-square test was used to analyse the relationship.

**Results:** Among our patients, 54% consumed alcohol. Among the 329 patients, 117 (35.5%) had fatal injuries. There was a significant association between alcohol consumption and victimization (χ² = 12.84, p < 0.0003). An association between alcohol intake and accident outcomes (fatal and nonfatal) among drivers was observed (χ² = 29.88, p < 0.00001). There was a significant association between alcohol intake and accident outcomes (fatal and nonfatal) among nondrivers (χ² = 6.806, p < 0.009).

**Conclusion: ** Accidents involving motor vehicles were more prevalent among younger age groups and males. To increase the effectiveness of alcohol prevention interventions, a multifaceted strategy focusing on these dimensions should be implemented. The incidence of road traffic accidents can be reduced by emphasizing the use of seat belts, avoiding the consumption of alcohol by drivers, and ensuring that roads are adequately lit, all of which can reduce the incidence of road traffic accidents.

## Introduction

Road Traffic Injuries (RTIs) are a significant public health issue and persist as a primary cause of mortality among those aged 15 to 49 years, resulting in roughly 1.2 million fatalities each year globally^1^. In India, the burden of road traffic accidents continues to rise. According to the Ministry of Road Transport and Highways, a total of 4,12,432 road accidents were reported in 2021, resulting in 1,42,163 fatalities and more than 3.8 lakh injuries^2^.

Various variables influence the incidence and severity of road traffic accidents, including excessive velocity, insufficient compliance with traffic laws, substandard road infrastructure, and impaired driving capabilities. Alcohol use is a recognised risk factor, negatively impacting judgement, response time, coordination, and risk perception. Prior research indicates that alcohol consumption is associated with a significant percentage of traffic-related injuries and deaths, especially among young adult males^3, 4,5^.

Developing countries account for more than 80% of global road traffic accident deaths and injuries, reflecting disparities in enforcement, awareness, and preventive strategies^6,8^. In India, rapid urbanisation, increased vehicle density, and easy accessibility to alcohol further exacerbate the problem^7^. Pondicherry, in particular, has been recognised as a region with relatively high alcohol consumption, making it an important setting for examining alcohol-related road traffic injuries.

This study was designed to investigate the relationship between recent alcohol intake and road traffic accident outcomes among patients at a tertiary care hospital in Pondicherry, India.

## Materials and methods

### Study design and settings

This was a cross-sectional, hospital-based observational study conducted in the Emergency Department of Indira Gandhi Medical College & Research Institute (IGMC&RI), a 1000-bed tertiary care teaching hospital located in Puducherry, India. The hospital functions as a principal referral site for trauma care in the region. The study was conducted from 25 May 2018 to 22 August 2018.

### Study population and eligibility criteria

#### Inclusion criteria

- Patients aged 15 years and above.
- Patients involved in Road Traffic Accident presenting to the emergency department during the study period.
- Patients who were clinically stable and able to participate in the study either directly or through a legally authorised representative.

#### Exclusion criteria

- Individuals who were brought dead or declared dead on arrival.
- Patients with severe injuries, operationally defined as those requiring immediate life-saving interventions, emergency surgical procedures, mechanical ventilation, or intensive resuscitation, where participation could interfere with urgent care.
- Patients for whom informed consent or proxy consent could not be obtained.

### Operational definition

- Road traffic accidents: For research purposes, a road traffic accident is defined as any collision on a road involving multiple objects, one of which must be a moving vehicle of any kind.
- Drivers: “The person in the vehicle who was driving or planning to drive at the time of the accident”.
- Pedestrian: “Any person in an accident who was walking on the road and not in or on a vehicle at the time of the accident”.
- Passengers: “A person who is travelling in a car, bus, train, plane, etc., but who is not driving it.”
- Alcohol consumption (exposure): “Presence or absence of recent alcohol intake proximate to the time of injury, determined using breath analyser or saliva alcohol testing. Alcohol exposure was treated as a binary variable (yes/no)”.
- Nondrivers: Nondrivers include passengers and pedestrians.

### Consent procedures

Informed consent was obtained from all eligible par prior to data collection. For conscious and oriented patients, written informed consent was obtained directly after explaining the purpose and procedures of the study.

For patients who were temporarily unconscious, disoriented, or unable to provide consent at presentation, consent was obtained from a legally authorized representative or accompanying family member, in accordance with institutional ethical guidelines. In such cases, patient participation was limited to non-invasive data collection and screening procedures. Patients who did not regain capacity or for whom proxy consent could not be obtained were excluded from the study.

No study procedures were conducted that interfered with emergency medical care, and participation was completely voluntary.

### Data collection

The study involved patients aged 15 years and older who presented to the Emergency Department of Indira Gandhi Medical College & Research Institute as a result of road traffic injuries during the study period. Individuals who demonstrated clinical stability and were capable of engaging in the interview process, either personally or via a legally authorised representative, qualified for inclusion in the study.

Individuals presenting with severe injuries were operationally characterised as those requiring immediate life-saving interventions, emergency surgical procedures, mechanical ventilation, or intensive resuscitation, wherein their involvement in the interview or alcohol testing could disrupt emergency medical care. The patients in question were excluded from the study.

Individuals who were pronounced dead upon arrival or brought in deceased were excluded from the study. This exclusion was necessary due to the study’s reliance on interview-based data collection and bedside alcohol screening, which could not be conducted ethically or practically in these circumstances.

Alcohol testing was conducted upon presentation to the emergency department utilising either a breath analyser or a saliva alcohol test, contingent upon the clinical condition and the patient’s level of cooperation. Breath analyser testing was conducted on subjects who were alert, cooperative, and exhibited stable respiratory patterns, thereby facilitating sufficient breath sampling. Saliva alcohol testing was employed for patients who were unconscious, uncooperative, or experiencing respiratory distress, conditions under which breath sampling was not practical. Both methods were utilised as rapid screening instruments for the assessment of recent alcohol consumption in an emergency context, rather than for the quantitative determination of blood alcohol concentration. Alcohol screening was performed using the Alco-Sensor FST Breathalyser, and saliva testing was conducted with the AlcoScreen Saliva Alcohol test. According to the manufacturer’s guidelines, breath alcohol readings indicating a blood alcohol concentration (BAC) of ≥0.03 g/dL (30 mg/100 mL) were classified as positive for recent alcohol consumption, while readings below this threshold were considered negative. BAC levels of ≥0.02% (approximately 20 mg/dL) in saliva were also classified as positive for recent alcohol consumption.A positive result was interpreted as indicative of recent alcohol consumption occurring close to the time of injury. The data collection period spanned from May 25, 2018, to August 22, 2018. A semi-structured questionnaire was employed for data collection in the study. Sociodemographic domains relevant to the context of the study were incorporated into the questionnaire. The data were obtained subsequent to the acquisition of informed consent from the patients. Data were collected by two qualified medical doctors. Data were collected using an electronic tablet through an open data kit (ODK).

### Data analysis

Data were analysed using IBM SPSS version 16. Descriptive statistics were used to summarise variables as frequencies and percentages. Associations between categorical variables, including alcohol consumption, driver status, and accident outcomes, were assessed using the Chi-square test.

The analysis was limited to bivariate comparisons, and no multivariable regression analysis was performed. Therefore, potential confounding factors such as age, sex, role in the accident (driver or nondriver), and environmental conditions were not adjusted for. A p-value of < 0.05 was considered statistically significant.

## Results

### Social and demographic variables

Among our 329 patients, nearly 33% were 36–45 years, 24% were 26–35 years, and 3.42% were older than 65 years. Nearly 75% of them are males, and 25% of them are females. Almost 71% of the accidents occurred in urban areas, and 29% occurred in rural areas. Among our patients, 73% were drivers, and 27% were nondrivers. Among our patients involved in Road Traffic Accident, 54% consumed alcohol, whereas 46% did not consume alcohol. Nearly 36% of them had fatal accidents. (Table 1).

**Table 1:** Social and demographic variables.(n = 329).

Variables		*n*	%
Age	15–25	61	18.45
	26–35	81	24.55
	36–45	109	33.21
	46–55	49	15.07
	56–65	18	5.3
	Above 65	11	3.42
Gender	Male	247	75.08
	Female	82	24.92
Area	Rural	95	28.88
	Urban	234	71.12
Patients involved in Road Traffic Accident	Drivers	241	73.25
	Non-Drivers	88	26.75
Alcohol consumption	Yes	177	53.79
	No	152	46.2
Fatal accidents	Yes	117	35.56
	No	212	64.44

### Association of alcohol consumption between drivers and nondrivers

The distribution of alcohol consumption among drivers and nondrivers is presented in Table 2. Among drivers, 144 (43.77%) tested positive for alcohol, whereas 97 (29.48%) tested negative. Among nondrivers, 33 (10.03%) tested positive and 55 (16.72%) tested negative.

**Table 2:** Association of alcohol consumption between drivers and nondrivers.(n = 329).

Alcohol consumption	Drivers	Non-drivers	Total	
Yes	144 (43.77%)	33 (10.03%)	177 (53.80%)	χ2 = 12.84 *P* < 0.0003
No	97 (29.48%)	55 (16.72%)	152 (46.20%)	
Total	241 (73.25%)	88 (26.75%)	329 (100%)	

A statistically significant association was observed between alcohol consumption and driver status (χ² = 12.84, *p* < 0.0003).

### Association of alcohol consumption with fatal and non-fatal accidents among drivers

Table 3 shows the distribution of alcohol consumption according to accident outcome among drivers. Among drivers who tested positive for alcohol, 79 (32.78%) sustained fatal injuries and 65 (26.75%) sustained non-fatal injuries. Among drivers who tested negative, 19 (7.88%) sustained fatal injuries and 78 (32.37%) sustained non-fatal injuries.

**Table 3:** Association of alcohol consumption with fatal and non-fatal accidents.(n = 241).

Alcohol intake	Drivers		Total	
	Fatal	Non-fatal		
Yes	79 (32.78%)	65 (26.75%)	144(59.75%)	χ2 = 29.88 *P* < 0.00001
No	19 (7.88)	78 (32.37%)	97 (40.25%)	
Total	98 (40.66%)	143 (59.34%)	241 (100%)	

The association between alcohol consumption and accident outcome among drivers was statistically significant (χ² = 29.88, *p* < 0.00001).

### Association of alcohol consumption with fatal and non-fatal accidents among nondrivers

Among nondrivers, alcohol consumption and accident outcomes are shown in Table 4. Twelve (13.64%) alcohol-positive nondrivers sustained fatal injuries, while 21 (23.86%) sustained non-fatal injuries. Among alcohol-negative nondrivers, 7 (7.95%) sustained fatal injuries and 48 (54.55%) sustained non-fatal injuries.

**Table 4:** Association of alcohol consumption with fatal and nonfatal accidents among nondrivers.(n = 88).

Alcohol intake	Non-drivers		Total	
	Fatal	Non-fatal		
Yes	12 (13.64%)	21 (23.86%)	33 (37.50%)	χ2 = 6.806 *P* < 0.009
No	7 (7.95%)	48 (54.55%)	55 (62.5%)	
Total	19 (21.59%)	69 (78.41%)	88 (100%)	

A statistically significant association was observed between alcohol consumption and accident outcome among nondrivers (χ^2^ = 6.806, *p* < 0.009).

## Discussion

The findings of the present study demonstrate statistically significant associations between recent alcohol consumption and road traffic accident outcomes. However, due to the cross-sectional design and the use of bivariate statistical analysis, these associations should not be interpreted as causal relationships. The absence of multivariable analysis limits the ability to account for potential confounding variables that may independently influence accident severity and fatality.

The sociodemographic profile of the study patients who underwent RTI included 75% more males than females (25%), similar to the findings of various other studies. The age group with the highest proportion of injuries consist of young people (15–45 years of age) in the current study as a result of risky driving behaviours^4^. The results above show the most patients involved in road-traffic accidents are men because men are more likely to drive, and younger people today are more likely to drive in dangerous ways.

In our study, 71% of the patients involved in road-traffic accidents were drivers from urban areas, and among the total patients involved in road-traffic accidents, 53.7% were intoxicated from alcohol consumption. Similar to the present study, a study conducted by Athanasia et al. in Greece included 40% of patients involved in road-traffic accidents who were found to be positive for alcohol use^9^. In contrast, the results of Jha Na et al., and Mohan et al. revealed that 14–19% of patients involved in road-traffic accidents used alcohol^10,11^.

According to a study by Ananthanarayan et al., in urban India, alcohol intoxication accounts for approximately 29.4% of cases^12^. This slightly greater percentage of drivers with alcohol use may be due to the greater frequency of vehicles in cities, increasing the likelihood of accidents, with Pondicherry being the heart of the city with increased access to alcohol, the results revealed a greater proportion of driver patients involved in road-traffic accidents with alcohol intoxication.

In addition, Mishra et al.^13^ revealed that 46.3% of drivers were intoxicated at the time of an RTA in their study; however, Mohan et al.^10^ concluded that this higher percentage can also depend on the testing procedure that has been used to determine the alcohol percentage, i.e., through breath/blood reports. Research by Honkanen^14^ and colleagues involved 201 drivers. Blood alcohol levels were assessed. They concluded that the most potent risk factor for injuries from traffic accidents was alcohol consumption. Alcohol use was detected in 15% of the patients.

According to the findings of the present study, 33% of all drivers who consumed alcohol during an accident sustained fatal injuries, which is significantly lower than the findings of Mohan and colleagues^10^. Furthermore, in a systematic analysis, Das et al. reported that numerous studies in India have linked alcohol intoxication to 6%–48% of fatalities and 2%–33% of injuries from RTIs^15^. While considering nonfatal injuries associated with alcohol consumption, 26% were nonfatal, which is in accordance with the findings of Peden et al.^16^. Therefore, there was a significant association (*p* < 0. 00001) between alcohol intoxication and the prevalence of fatal injuries. These findings are consistent with the present study, which observed a statistically significant association between alcohol positivity and a higher proportion of fatal accident outcomes among drivers.

### Limitations

This study possesses specific limitations that warrant acknowledgement. Alcohol exposure was quantified as a binary variable, excluding information regarding the amount consumed, type of alcoholic beverage, duration of consumption, habitual drinking patterns, or the time elapsed between the last alcohol intake and the occurrence of the accident. The absence of measurements for blood alcohol concentration levels restricts the capacity to evaluate the dose-dependent effects of alcohol on the severity of injuries and the associated risk of fatality. Furthermore, alcohol testing was conducted exclusively on cooperative patients, potentially resulting in an underestimation of alcohol involvement in cases of severely injured or unconscious patients involved in road-traffic accidents. It is essential to acknowledge these limitations when interpreting the findings, and it is advisable for future studies to incorporate quantitative toxicological analysis.

Furthermore, the exclusion of individuals who were deceased upon arrival, as well as those presenting with severe injuries necessitating immediate life-saving interventions, may have resulted in selection bias. This could potentially lead to an underestimation of the actual burden of alcohol involvement in the most severe and fatal road traffic accidents. Methodological constraints of this nature are frequently observed in hospital-based observational studies carried out within emergency care environments.

The research is additionally constrained by the lack of multivariable statistical modelling. The observed associations may be influenced by unmeasured variables in the absence of adjustments for potential confounders, including age, sex, driving role, use of protective devices, and environmental factors. As a result, the findings indicate unadjusted associations instead of independent effects of alcohol consumption on accident outcomes. The limitations identified are intrinsic to hospital-based cross-sectional studies and must be taken into account when interpreting the results.

## Conclusion

The present hospital-based study identified statistically significant associations between recent alcohol consumption and road traffic accident outcomes among both drivers and nondrivers. Alcohol positivity was more frequently observed among young male drivers, and a higher proportion of fatal outcomes was noted among alcohol-positive individuals.

Given the cross-sectional design, qualitative assessment of alcohol exposure, and absence of multivariable adjustment, these findings should be interpreted as associations rather than causal relationships. Nonetheless, the results underscore the continued public health relevance of alcohol use in road traffic injuries. Strengthening enforcement of drunk-driving laws, improving public awareness, and integrating routine alcohol screening in emergency settings may contribute to reducing alcohol-related road traffic injuries. Further studies incorporating quantitative toxicological analysis and multivariable modelling are warranted.

## Data Availability

The data that support the findings of this study are available from Emergency Department at Indira Gandhi Medical College & Research Institute (IGMC&RI) but restrictions apply to the availability of these data, which were used under license for the current study, and so are not publicly available. Data are however available from the authors upon reasonable request and with permission of Emergency Department at Indira Gandhi Medical College & Research Institute (IGMC&RI).
